# Cas13d-mediated multiplex RNA targeting confers a broad-spectrum resistance against RNA viruses in potato

**DOI:** 10.1038/s42003-023-05205-2

**Published:** 2023-08-17

**Authors:** Xiaohui Zhan, Wenting Liu, Bihua Nie, Fengjuan Zhang, Jiang Zhang

**Affiliations:** 1 State Key Laboratory of Biocatalysis and Enzyme Engineering, Hubei Hongshan Laboratory, School of Life Sciences, Hubei University, Wuhan, 430062 China; 2 Key Laboratory of Potato Biology and Biotechnology, Ministry of Agriculture and Rural Affairs, Key Laboratory of Horticultural Plant Biology, Ministry of Education, Huazhong Agricultural University, Wuhan, 430070 China; 3 Shenzhen Branch, Guangdong Laboratory of Lingnan Modern Agriculture, Key Laboratory of Synthetic Biology, Ministry of Agriculture and Rural Affairs, Agricultural Genomics Institute at Shenzhen, Chinese Academy of Agricultural Sciences, Shenzhen, 518000 China

**Keywords:** Molecular engineering in plants, CRISPR-Cas systems

## Abstract

CRISPR-Cas systems endow the bacterial and archaeal species with adaptive immune mechanisms to fend off invading phages and foreign plasmids. The class 2 type VI CRISPR/Cas effector Cas13d has been harnessed to confer the protection against RNA viruses in diverse eukaryotic species. However a vast number of different viruses can potentially infect the same host plant resulting in mixed infection, thus necessitating the generation of crops with broad-spectrum resistance to multiple viruses. Here we report the repurposing of CRISPR/Cas13d coupled with an endogenous tRNA-processing system (polycistronic tRNA-gRNA, PTG) to target the multiple potato RNA viruses. Expression of Cas13d and four different gRNAs were observed in transgenic potato lines expressing the Cas13d/PTG construct. We show that the Cas13d/PTG transgenic plants exhibit resistance to either PVY, PVS, PVX or PLRV alone or two/three viruses simultaneously by reducing viral accumulation in plant cells. In sum, our findings provide an efficient strategy for engineering crops that can simultaneously resist infection by multiple RNA viruses.

## Abstract (web summary)

CRISPR/Cas13d coupled with an endogenous tRNA-processing system can be used to engineer resistance to multiple RNA viruses in potato.

## Introduction

Plant viruses are obligate intracellular parasites replicating themselves by using the molecular machinery of their hosts. Plant viral diseases can cause severe yield loss and continuously threaten crop production worldwide^1–3^. Diverse approaches of controlling RNA viruses in plants have been attempted including conventional breeding, genetic engineering and RNA interference^4^. However, due to the high rate of mutation and recombination of viruses, the virus-resistant plants could be overcome in a few years^5^. In addition, many plant viruses evolve and often develop various counter-defense mechanisms, such as viral suppressors of RNA silencing, nullifying the current antiviral approaches^6,7^.

Potato (*Solanum tuberosum* L.) is the world’s most important non-grain crop^8^ which is also sensitive to various biotic and abiotic stresses. Viruses are among the most significant biotic constraints in potato production. Potato is clonally propagated by planting tubers, further increasing the risk of virus infection. Until now, more than 50 different viruses have been reported to be able to infect potatoes worldwide. Among them, potato leaf roll virus (PLRV), potato virus Y (PVY), potato virus X (PVX) and potato virus S (PVS) have been recognized as the major potato viruses^3,9^. These four RNA viruses are all positive-sense single-stranded RNA (+ssRNA). PLRV is a type species of the genus *Polerovirus*, belonging to the family *Solemoviridae*^10,11^. An intriguing feature of PLRV is the phloem restriction. It can be delivered into phloem tissues by aphids, agro-inoculation or grafting, while not be transmitted by mechanical inoculation. Besides, PVY is a member of the genus *Potyvirus* in the family *Potyviridae* and is transmitted by aphids in a non-persistent manner or through potato tubers^12^. PVY is prone to high mutation and recombination rates, existing as a complex of strain groups, or variants^13,14^. On the other hand, PVS belongs to genus *Carlavirus* in the family *Flexiviridae*^15,16^. PVS can be transmitted by plant contact or several aphid species in a non-persistent manner^17^. Apart from this, PVX is a type species of the genus *Potexvirus* in the family *Alphaflexiviridae*^18^. The symptoms induced by PVY or PLRV are visible when infected with single virus, such as mosaic, mottled and crinkled leaves as well as leaf and vein necrosis in PVY infection^14,19^, and leaf rolling and stunting in PLRV infection^10^. Upon infection with a specific virus, PVY and PLRV are among the most damaging viruses of potato worldwide^9,20,21^. However, in the field, potato is usually challenged by multiple species of RNA viruses, which result in more significant loss of tuber quality and quantity^22^. For example, PVX or PVS causes varying degrees of tuber yield losses in the single infection, while the symptom and production loss can be more severe in the mixed infection^3,23^.

The CRISPR/Cas system (Clustered Regularly Interspaced Short Palindromic Repeats associated proteins) is emerging as a promising tool for gene editing and sequence-specific regulation of gene expression^24–26^. The Cas9 and Cas12 of Class II were employed to combat DNA viruses in eukaryotes^27,28^. The type VI-A effector Cas13a (previously known as C2c2) has been characterized for cleaving single-stranded RNA^29^ and designed to interfere with viral RNA replication against the turnip mosaic virus via transient transformation assays^30^. Consistent with this, the CRISPR/Cas13a system can be engineered to target PVY genome and confer resistance to PVY in the transgenic potato plants by the stable transformation^31^. Similarly, CRISPR/Cas13a system could enable the plant to acquire potent defense against viral infection in both tobacco and rice plants^32^.

Recently, a new Cas13 subtype, type VI-D (Cas13d), has been identified. Cas13d effector is relatively smaller compared to previously reported subtypes. Moreover, Cas13d was shown to have more robust RNA virus interference applications compared to LwaCas13a and PspCas13b variants in *Nicotiana benthamiana*^33^, making it suitable for diverse RNA viruses targeting or detection^34^.

It has been demonstrated that numerous gRNAs can be generated by employing the endogenous tRNA-processing system of plant cells, enabling the simultaneous targeting of multiple genomic loci^35^. To produce multi-virus-resistant plants, we sought to develop a robust multiple RNA-targeting CRISPR/Cas13d system conferring resistance to different RNA viruses. We show that CRISPR/Cas13d assembled with a *PTG* gene can reduce the multiplexed viral accumulation in transgenic potato plants, providing a new strategy for conferring broad-spectrum resistance to viral diseases in crops.

## Result

### Engineering a tRNA-processing System for Producing Four gRNAs

**Fig. 1:**
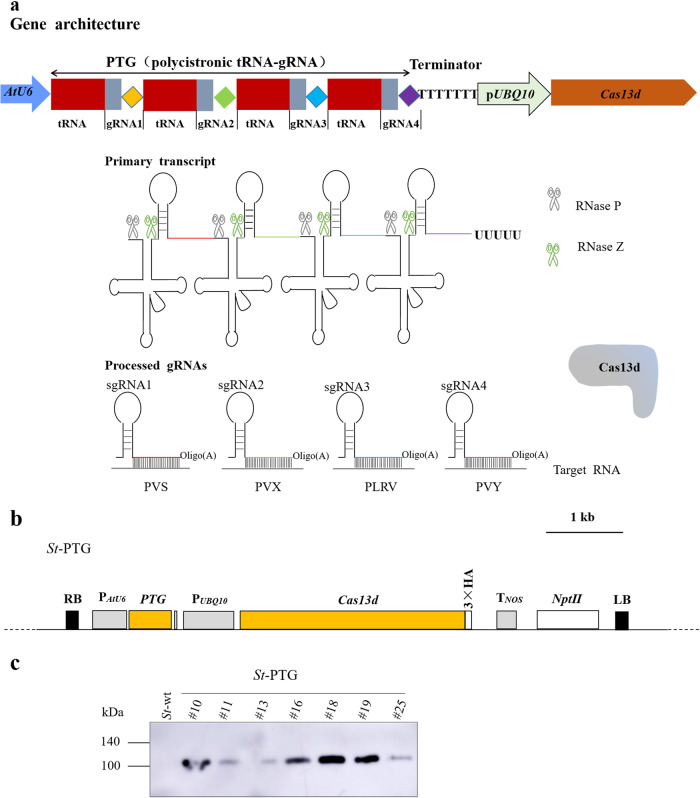
Engineering of the endogenous tRNA-processing system for multiplex RNA targeting with CRISPR/Cas13d. **a** Schematic depiction of the Cas13d/PTG system for simultaneously targeting multiple viruses. The synthetic *PTG* consists of tandemly arrayed tRNA-gRNA units, with each gRNA containing a sequence-specific spacer designed for targeting one RNA virus genome (labeled as diamonds with different colors) and conserved gRNA scaffold (gray rectangle). The tRNAs are shown as red rectangles. The primary transcript of *PTG* is cleaved by endogenous RNase P and RNase Z (labeled as scissors) to release mature gRNAs and tRNA (lines of cloverleaf structure). The excised mature gRNAs containing four individual gRNA (PVS-sgRNA, PVX-sgRNA, PLRV-sgRNA and PVY-sgRNA), directing Cas13d to four RNA virus genomes. The oligo(A) motif of the sgRNA serves as a signal for nuclear export. **b** Map of the T-DNA locus in transgenic potato lines transformed with a construct for expression of Cas13d/PTG (*St*-PTG). *PTG* contains four gRNAs targeting four RNA virus genomes as shown in (a). 3 × HA: hemagglutinin epitope. **c** Analysis of the Cas13d expression in the transgenic potato lines by western blotting. Anti-HA antibody was used to detect the expression of HA-tagged Cas13d protein.

To target multiple RNA viruses simultaneously, we constructed a binary vector (pPTG) harboring a synthetic *Cas13d* gene (fusion with a 3 × HA tag sequence) from *Ruminococcus flavefaciens* XPD3002 (CasRx) and a polycistronic tRNA-gRNA (*PTG*) gene for simultaneous production of four gRNAs (Fig. 1a; Supplementary Fig. 1), targeting the *CP* genes of four potato viruses (PVS, PVY, PLRV and PVX). The expression of *Cas13d* and *PTG* genes is driven by the *UBQ10* (*Arabidopsis* ubiquitin-10) and *AtU6* promoters, respectively (Fig. 1b). Each sgRNA contains an oligo (A)-rich tail, which serves as a signal for nuclear export^36^.

We next introduced the pPTG construct into potato plants by stable *Agrobacterium*-mediated transformation. Transgenic lines were selected on kanamycin-containing plant regeneration medium. Over 15 independent transgenic lines were obtained. The expression of Cas13d was determined by western blotting (Fig. 1c). Four gRNAs transcribed from the *PTG* genes were verified by qRT-PCR (Supplementary Fig. 2a–d). Two independent transgenic lines (*St*-PTG#18 and *St*-PTG#19) with relatively higher transgene expression were chosen for further experiments. Furthermore, phenotypes of transgenic lines growing on synthetic medium and soil were screened. We found that the transgenic plants expressing Cas13d/PTG were indistinguishable from wild-type plants under mixotrophic and autotrophic growth conditions (Supplementary Fig. 4a). The height, the number of aerial branches and stems of transgenic plants were similar to those of wild-type potato plants (Supplementary Fig. 4b–d).

### Cas13d/PTG-mediated interference with PLRV, PVY, PVS or PVX

**Fig. 2:**
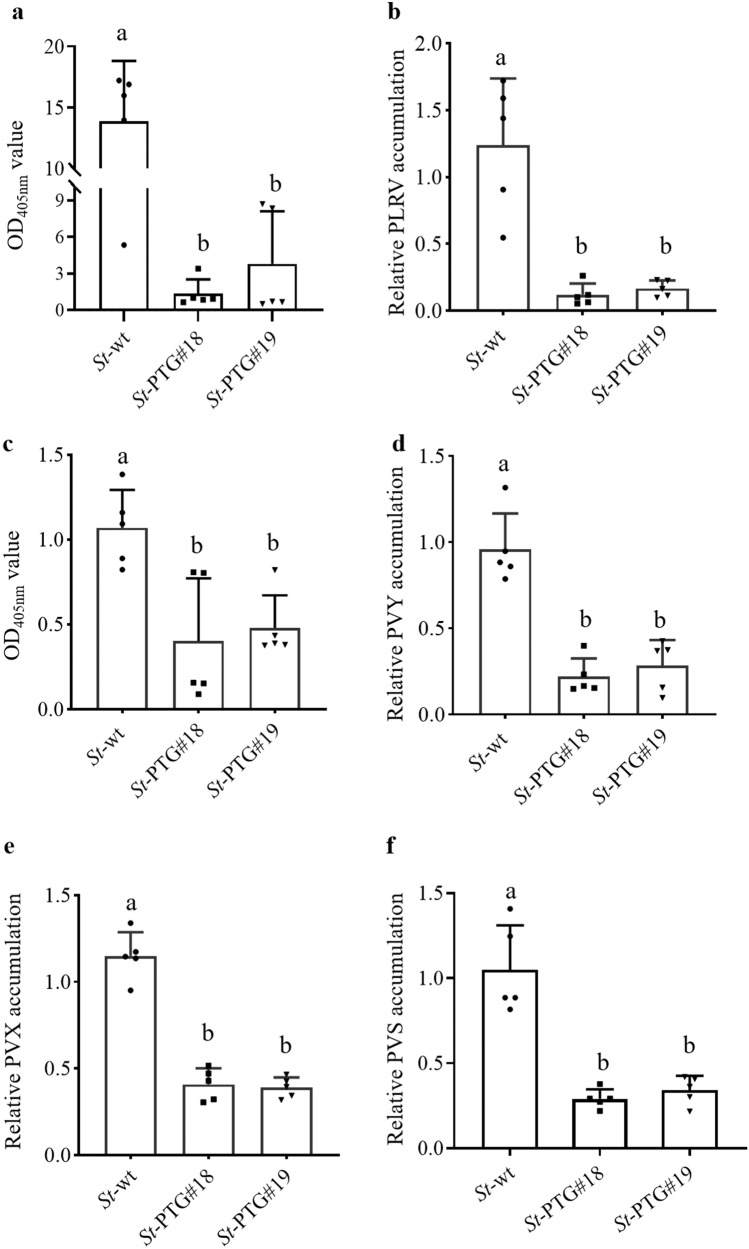
Resistance of transgenic potato plants expressing Cas13d/PTG to four individual RNA viruses. PLRV accumulation in transgenic potato plants was assessed at 15 dpi by ELISA (**a**) and qRT-PCR (**b**). PVY^O^ accumulation in transgenic plant was assessed at 25 dpi by ELISA (**c**) and qRT-PCR (**d**). PVX (**e**) and PVS (**f**) accumulation in transgenic potato plants was assessed at 20 dpi by qRT-PCR. Data are means ± SD, and represent five biological replicates (*n* = 5). The letters above the bar indicate the significant differences as determined by one-way ANOVA (*P* < 0.05).

We first determined whether engineering of Cas13d/PTG system in potato plants could confer the resistance against individual RNA virus. To this end, transgenic (*St*-PTG#18 and *St*-PTG#19) and wild-type (*St*-wt) plants were challenged with PLRV or PVY^O^. Accumulation of PLRV or PVY^O^ was assessed at 15 and 25 days post-inoculation (dpi) by enzyme-linked immunosorbent assays (ELISA) and qRT-PCR assays, respectively. Resistance of *St*-PTG transgenic plants to PLRV or PVY^O^ was evidenced by a strongly reduced accumulation of PLRV and PVY^O^ in the systemic leaves (Fig. 2a–d). Similarly, transgenic and wild-type (*St*-wt) plants were exposed to PVS or PVX and the virus accumulation was determined at 15 and 20 dpi by qRT-PCR. We showed that, compared to wild-type control plants, Cas13d/PTG expression strongly inhibited PVS or PVX accumulation in the systemic leaves of the virus-infected transgenic plants (Fig. 2e, f, Supplementary Fig. 5). To further evaluate the resistance level, the *S. chacoense* accession 40-3 containing the *Ry*_*chc*_ gene that could confer extreme resistance to PVY^37^ was chose for comparison. Although the resistance level to PVY of Cas13d/PTG transgenic plants was relatively lower than that of PVY-resistant line 40-3, they were additionally resistant to PLRV, PVS and PVX (Supplementary Fig. 6). These results demonstrate that Cas13d/PTG transgenic plants could reduce the accumulation of four individual viruses, respectively.

### Cas13d/PTG exhibits multiplexed virus-targeting activity

**Fig. 3:**
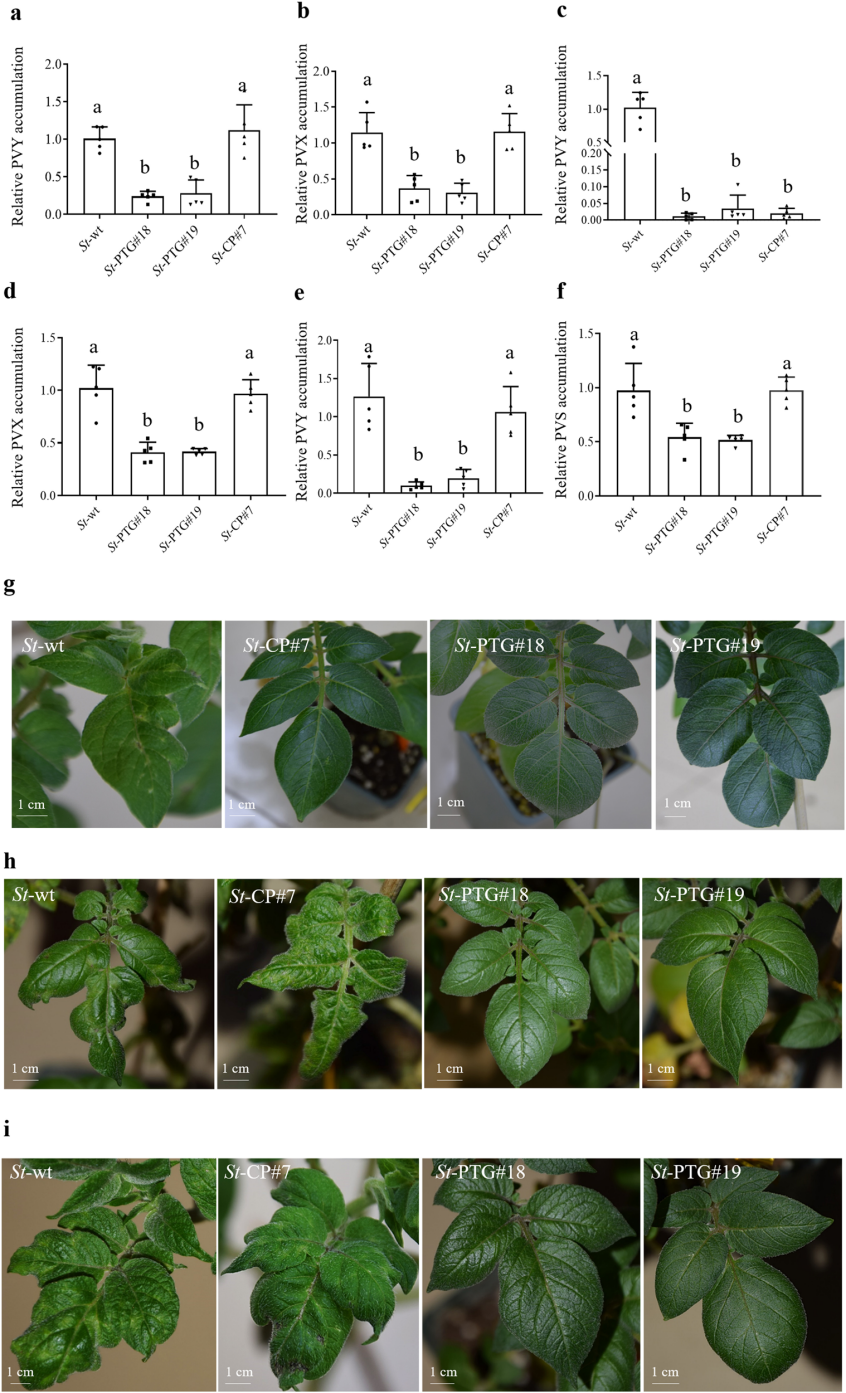
Resistance of transgenic potato plants expressing PTG to the mixtures of two RNA viruses. In the mixed infection with PVY/PVX mixture, PVY (**a**) and PVX (**b**) accumulation in the indicated transgenic plants at 25 dpi was determined by qRT-PCR. Accumulation of infection with individual PVY (**c**) and PVX (**d**) in the indicated transgenic plant at 25 dpi was determined by qRT-PCR. Similarly, the mixture of PVY/PVS was exposed to wild type and transgenic plants. PVY (**e**) and PVS (**f**) accumulation in the indicated transgenic plant at 25 dpi were shown by qRT-PCR. A transgenic line only targeting the *CP* gene of PVY (*St*-CP#7) (Zhan et al., 2019) was served as a control. Data represent five biological replicates (*n* = 5). The letters above the bars indicate the significant differences as determined by one-way ANOVA (*P* < 0.05). Disease symptoms in response to individual infection (only PVY) (**g**) and mixed infection (PVY/PVX) (**h**). Symptoms of transgenic and wild-type potato plants challenged with mixed viruses (PVY/PVS) at 25 dpi (**i**).

**Fig. 4:**
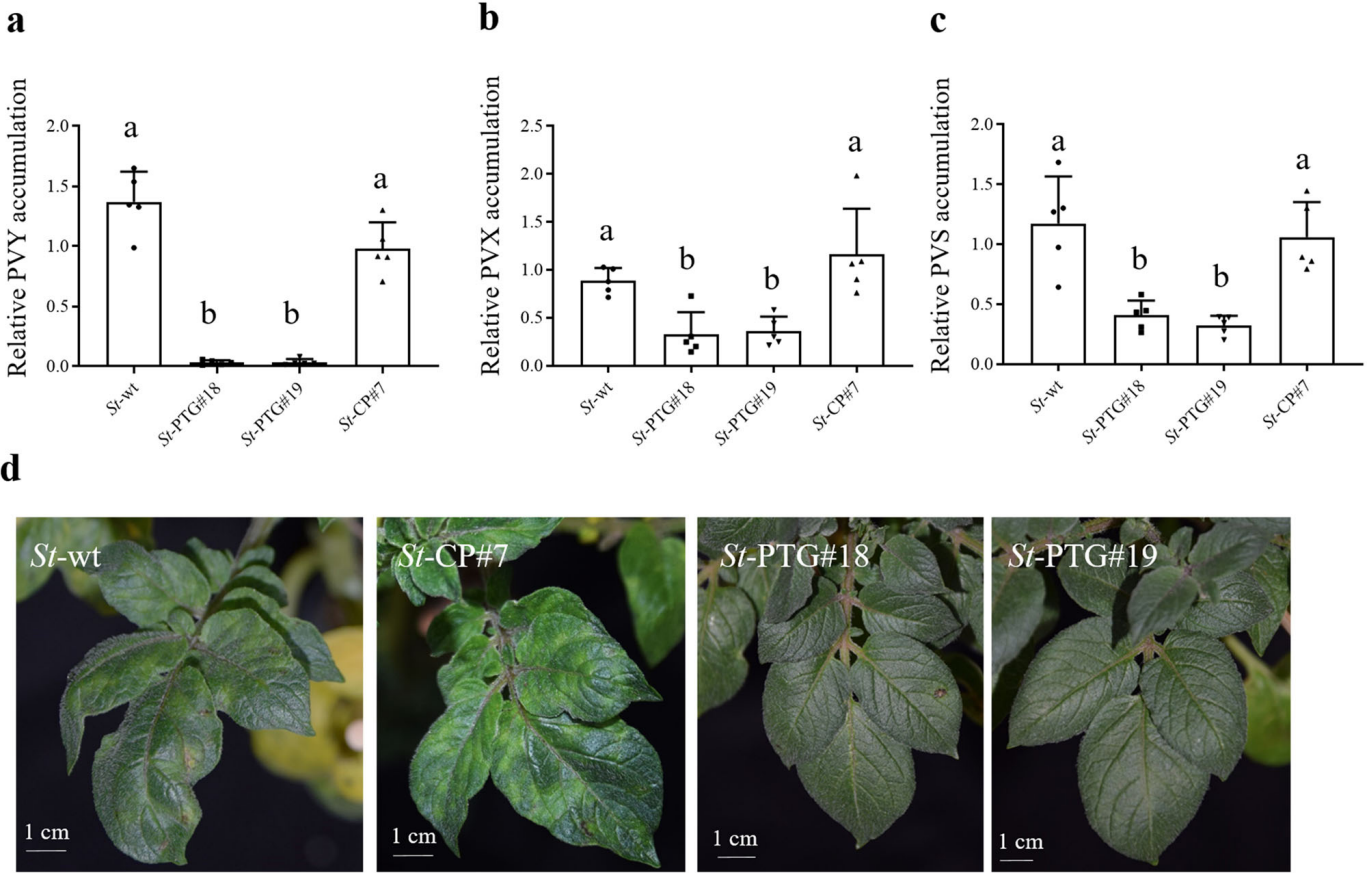
The response to mixed infection of three RNA viruses in transgenic potato plants expressing Cas13d/PTG. Transgenic and wild type potato plants were challenged with the mixture of PVY/PVX/PVS. The lower virus accumulation of PVY (**a**), PVX (**b**) and PVS (**c**) were verified in transgenic plants expressing Cas13d/PTG (*St*-PTG#18 and *St*-PTG#19) by qRT-PCR at 25 dpi. Data are means ± SD, and represent five biological replicates (*n* = 5). The significant differences were determined by one-way ANOVA (*P* < 0.05). **d** The symptoms such as mosaic, mottled and crinkled leaves were displayed in wild type and *St*-CP#7 transgenic plants, while transgenic plants expressing Cas13d/PTG (*St*-PTG#18 and *St*-PTG#19) had no obvious symptom.

To investigate whether the Cas13d/PTG is capable of targeting multiple RNA viruses simultaneously, we performed mechanical inoculation with mixed PVY/PVX to infect transgenic (*St*-PTG#18 and *St*-PTG#19) and wild-type (*St*-wt) plants. A PVY-resistant line (*St*-CP#7) expressing a single guide RNA targeting the *CP* gene of PVY^31^ (Supplementary Fig. 3a) was used for comparison. The virus titer was assessed and evaluated at 25 dpi by qRT-PCR. We found that transgenic plants expressing Cas13d/PTG exhibited significantly reduced accumulation of both PVY and PVX compared to wild-type and *St*-CP transgenic plants (Fig. 3a, b). When infected with individual PVY or PVX, transgenic plants expressing Cas13d/PTG can also interfere with PVY and PVX replication. In contrast, the PVY-resistant line (*St*-CP#7) can only reduce the accumulation and disease symptoms of PVY, but not PVX (Fig. 3c, d, g). These results indicated engineering of Cas13d/PTG system in potato plants was capable of targeting two viruses simultaneously, while the transgenic plant expressing single guide RNA (*St*-CP#7) failed to attenuate the accumulation of mixed viruses (Fig. 3a). Moreover, the PVY accumulation in *St*-CP#7 transgenic plants was shown to be similar to those of wild-type plants when infected with mixed PVY/PVX, suggesting the mixture of PVY/PVX could breakdown the PVY resistance of transgenic plants expressing a single guide RNA. Next, we challenged the transgenic and wild-type plants with mixed PVS/PVY, a typical mixture in the field. The qRT-PCR assays were performed to detect the virus accumulation at 25 dpi. Similarly, we observed a lower accumulation of PVS and PVY in the Cas13d/PTG transgenic plants compared to wild-type and *St*-CP#7 transgenic plants (Fig. 3e, f). While severe mosaic, mottled and crinkled leaves were observed in the infected leaves of wild-type and *St*-CP#7 transgenic plants, almost no disease symptoms were observed in the Cas13d/PTG transgenic plants in the mixed infection of PVY/PVX or PVS/PVY (Fig. 3h, i). Finally, three mixed viruses infection of PVY/PVX/ PVS was also assayed. Similar to the observation of two mixed viruses, a significant decrease of PVY, PVX and PVS accumulation in Cas13d/PTG transgenic plants was verified compared to wild-type and *St*-CP#7 transgenic plants (Fig. 4a–c). The disease symptom was presented in the systemic leaves in wild-type and *St*-CP#7 transgenic plants, while no obvious symptom was observed in transgenic plants expressing Cas13d/PTG (Fig. 4d). Thus, these results indicated that the Cas13d/PTG system has a high potential to produce numerous gRNAs and target multiple virus genomes simultaneously.

## Discussion

In this study, we engineered a novel Cas13d/PTG system to efficient protection of transgenic potato plants from four RNA viruses either individually or simultaneously by producing four gRNAs from a single synthetic *PTG* gene (Figs. 1–4). Previous study has demonstrated the proof-of-concept of simultaneously expressing several gRNAs to interfere with PVX-GFP and TRBO-BFP RNA replication in the model plant *Nicotiana benthamiana* by the transient transformation assays^33^. Recently, the CRISPR/Cas13 has been reported to recover the RNA silencing activity in host cell by targeting SPCSV-*RNase3*, improving the SPVD resistance of sweet potato^38^. Differing from these findings, we confirmed the potential of the CRISPR/Cas13d system to confer the stable resistance to multiple important viral disease in potato plants by directly targeting multiple virus genomes (Figs. 3, 4).

It is worth highlighting that a PVY-resistant line (*St*-CP#7) can only reduce the accumulation of PVY (Supplementary Fig. 3a), but not PLRV, PVX and PVS (Supplementary Fig. 3b–d). Many groups of viruses (such as PLRV, PVY, PVS, PVM, PVX and PVA) have been found in the potato field^39^. The mixed infection of potato viruses (usually two or three different viruses) commonly occurred with diverse combinations^3,22^. For example, PVX, PVY and PVS are mostly found to make interactions, the percentage of PVX and PVY co-infection was equivalent with PVY and PVS co-infection in potato plants^40^. Our results indicated that the expression of four gRNAs (*St*-PTG) exhibited high interference with two or three mixed viruses. While interfere with the PVY accumulation when infected with PVY alone (Fig. 3c; Supplementary Fig. 3a), transgenic plant expressing a single gRNA (*St*-CP#7) has no effect on reduction of PVY accumulation in mixed infection (Figs. 3a, e, 4a). Mixed infection is often associated with an increase in symptom severity and virus accumulation in comparison with the plants infected by a single virus^40,41^. Thus the interference of PVY accumulation in mixed viruses could be covered by synergistic effects of two or three viruses in transgenic plant expressing a single gRNA, while Cas13d/PTG transgenic plants may outperform in the mixed infection by targeting two or three RNA viruses.

CRISPR/PTG system has been first applied in simultaneously expressing several gRNAs (up to eight) by employing an endogenous tRNA-processing system in rice protoplasts to efficiently guide Cas9 to multiple chromosomal targets^35^. We showed that the Cas13d/PTG system could exhibit targeting multiple potato virus genomes, suggesting the PTG technology could be adapted to wide organisms for multiple genome targeting. It would also be reasonable that the Cas13d/PTG system could target more than four different viruses. In this case, multiple gRNAs could be embedded in PTG system matching with different virus genomes. It was known that RNase P and RNase Z precisely recognize RNA substrates with tRNA-like structures^42^. Since there are number of tRNA genes with variable sequences, these could be embedded in *PTG* gene for generation of multiple gRNAs. In addition, the establishment of the Cas13d/PTG system in plants would provide a platform to dissect the functions of multiple endogenous gene at RNA levels.

To engineer multi-resistance to RNA viruses, multiplexed gRNA expression would be crucial. Therefore, the use of PTG would be necessary for engineering to resistance to multiple viruses. Except for the endogenous tRNA system used in this study, exogenous co-expression of Csy4 or self-cleaving ribozymes such as Hammerhead or HDV ribozymes flanking by each gRNA are another alternative methods to produce multiple gRNAs simultaneously^43–45^. These strategies would allow the expression of multiple gRNAs from a single transcript.

Although the expression of PVY-sgRNA in *St*-PTG transgenic plants was significantly lower compared to that of *St*-CP#7 transgenic plants (Supplementary Fig. 2d), the PVY accumulation levels in *St*-PTG transgenic plants were similar to those in *St*-CP#7 transgenic plants (Fig. 3c). Thus, besides conferring board-spectrum resistance to RNA viruses, PTG technology would also efficiently target single virus without impairing the RNA interfering efficiency. Moreover, although the resistance levels to PVY is lower in Cas13d/PTG transgenic plants compared to that in PVY-resistant line 40-3, the Cas13d/PTG transgenic plants can additionally generate stable broad-spectrum resistance to other RNA viruses (Supplementary Fig. 6a, c–d), while the PVY-resistant line 40-3 only specifically resistant to PVY (Supplementary Fig. 6b).

In sum, we developed a Cas13d/PTG system for engineering broad-spectrum resistance to multiple RNA viruses in potato. The Cas13d/PTG system enables programmable RNA virus interference for targeting either one virus alone or two/three mixed RNA viruses simultaneously, thereby extending the applicability of the CRISPR system to crop protection against multiple RNA viruses.

## Materials and methods

### Plant material and viral strains

Potato (*Solanum tuberosum* cv. Désirée) plants were grown under standard greenhouse conditions. Four virus isolates PVY^O^-FL (HM367075), PLRV (MT264739.1), PVX (AB056718.1) and PVS (KU896946) were maintained in tobacco or potato plants as inoculated hosts. *S. chacoense* accession 40-3 containing the *Ry*_*chc*_ gene that confers extreme resistance to PVY^37^ was grown under standard greenhouse conditions for comparison.

### Vector construction for potato transformation

*Cas13d* (*Ruminococcus flavefaciens* XPD3002, *CasRx*) gene sequence was codon optimized for expression in *S. tuberosum* (Supplementary Note 1). Four tandemly arrayed tRNA-gRNA units, with each gRNA targeting the *CP* genes (encoding virus coat protein) of four different RNA viruses (PVS, PVX, PLRV and PVY) (Supplementary Note 2), were designed and named as *PTG* gene. *Cas13d* and *PTG* genes were synthesized by GeneCreate (Wuhan, China). Subsequently, *PTG* gene with a poly(T) sequence (as a terminator) was cloned into under the *Arabidopsis U6* promoter using the restriction enzymes BsaI. *Cas13d* sequence was cloned as EcoRI/KpnI fragment into the similarly cut vector pJZH1^31^, generating plasmid pEV. *Arabidopsis U6* promoter was amplified using pJZH3 as template, and inserted as SacII/SbfI fragment into the pEV vector digested with SacII and SbfI, generating plasmid pXH9. Finally, the PTG sequence was cloned into pXH9 digested with BsaI, producing plasmid pPTG. Sanger sequencing was used to confirm the sequence accuracy of all the clones.

### Generation of the transgenic potato plants

*Agrobacterium tumefactions* strain GV3101 harboring the pPTG construct was employed to transform the potato plants (*S. tuberosum* cv. Désirée) using a protocol described previously^46^. Transgenic plants were screened by kanamycin (50 mg L^−1^) selection and initially tested for the presence of the transgene by PCR assays. *Cas13d* and gRNA expression levels were subsequently determined by qRT-PCR.

### Protein extraction and Western blot analysis

Total proteins were extracted from ~100 mg of leaf samples using protein extraction buffer (Na_2_S_2_O_5_, 50 mM; Tris-HCl, 125 mM, pH8.8; SDS, 1% (w/v); Glycerol, 10% (v/v)). Proteins were separated on an 8% polyacrylamide gel. Cas13d expression was detected by western blot analysis. An anti-HA antibody (ABclonal, Oxfordshire, UK) was used as primary antibody (1:3000 dilution). The signals on the membrane were visualized using the enhanced chemiluminescence substrate (SuperSignal West Pico; Pierce, Rockford, I L) following the manufacturer’s instructions.

### RNA extraction and qRT-PCR analysis of viral RNA genomes

Total RNA was extracted from virus-infected plants using the TRIzol reagent (Invitrogen, Carlsbad, CA) according to the manufacturer’s instructions. For virus expression analysis, cDNA was synthesized using reverse transcriptase and oligo(dT) primer (TaKaRa, Japan). qRT-PCR assays were carried out in a CFX96 Touch TM real-time PCR detection system (Bio-Rad, Hercules, CA) using SYBR Premix Ex Taq TM II (TaKaRa). Three technical replicates were performed for each biological replicate. The potato *TUBULIN2* gene was used as a reference. Primer sequences for qRT-PCR are listed in Supplementary Table 1.

### ELISA assay

Double-antibody sandwich enzyme-linked immunosorbent assays (DAS-ELISA) with virus-specific antibodies (Agdia, Elkhart, IN) were conducted to measure virus accumulation at the protein levels in the systemic leaves according to the manufacturer’s protocol^17^.

### Virus inoculation

To determine the response of transgenic plants to various viral strains or isolates, wild-type and transgenic plants were grown under long-day conditions (16-h light: 8-h dark) in the greenhouse at 20–25 °C. Aphid transmission was used for single virus infection except for PVX, which is generally considered transmitted by mechanical inoculation. Aphids were reared on virus-infected wild-type tobacco plants under controlled growth conditions at 20–25 °C. 25 adults were transferred onto wild-type and transgenic potato plants to transmit viruses from the aphids to the plants for 2 days, and then the aphids were removed.

For mechanical inoculation, five to eight plants of each line at the 6–8 leaf stage were inoculated with wild-type virus inocula or isolates (leaf extract: 1 g leaf tissue homogenized in 10 ml 0.1 M potassium phosphate buffer, pH 7.4) by mechanical wounding as described previously^47^. The mixed infection were also performed by mechanical inoculation using the inocula of the two or three viruses mixed at 1:1 or 1:1:1 titer.

### Statistics and reproducibility

The data for virus expression analysis including ELISA and qRT-PCR were analyzed using one-way analysis of variance (ANOVA) coupled with Tukey or Dunnett’s test for multiple comparisons. All the virus assays were repeated at least three independent experiments. Three technical replicates were performed for each biological replicate.

### Reporting summary

Further information on research design is available in the Nature Portfolio Reporting Summary linked to this article.

### Supplementary information

Supplementary Material

Description of Additional Supplementary Files

Supplementary Data 1

Reporting Summary

## Data Availability

All data generated or analyzed during this study are included in this article (and its supplementary information files). Uncropped and unedited blot/gel images is provided as Supplementary Fig. 7. Source data underlying figures are presented in Supplementary Data 1.
