# Supplementary material for: Cas13d-mediated multiplex RNA targeting confers a broad-spectrum resistance against RNA viruses in potato
Source: Commun Biol. 2023 Aug 17;6:855. doi: 10.1038/s42003-023-05205-2 (PMC10435558; doi:10.1038/s42003-023-05205-2)
Supplement: Supplementary file 1 — Supplementary Material [file 42003_2023_5205_MOESM1_ESM.pdf]

Supplemental Information for

Cas13d-mediated multiplex RNA targeting confers a broad-spectrum resistance against RNA viruses in potato

Xiaohui Zhan[a#], Wenting Liu [a#], Bihua Nie[b], Fengjuan Zhang[a*], Jiang Zhang[a,c*]

[a] State Key Laboratory of Biocatalysis and Enzyme Engineering, Hubei Hongshan Laboratory, School of Life Sciences, Hubei University, Wuhan 430062, China.

[b] Key Laboratory of Potato Biology and Biotechnology, Ministry of Agriculture and Rural Affairs, Key Laboratory of Horticultural Plant Biology, Ministry of Education, Huazhong Agricultural University, Wuhan 430070, China.

[c] Shenzhen Branch, Guangdong Laboratory of Lingnan Modern Agriculture, Key Laboratory of Synthetic Biology, Ministry of Agriculture and Rural Affairs, Agricultural Genomics Institute at Shenzhen, Chinese Academy of Agricultural Sciences, Shenzhen 518000, China.

This PDF file includes:

Supplementary Figures 1 to 7

Supplementary Note 1 and 2

Supplementary Table 1

* Corresponding authors: Zhang, J. (zhangjiang@hubu.edu.cn) Zhang, F.J. (zhangfengjuan159@163.com)

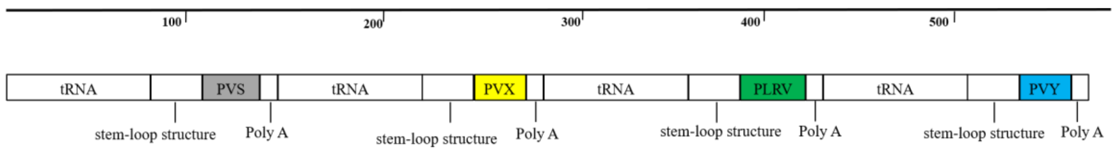

stem-loop structure : CCACCCCAATATCGAAGGGGACTAAAAC

Poly A : AAAAAAA

PVS-sgRNA : CTCTTGTGAGCGTTATGAGCTATCGTCT

PVX-sgRNA : TTCCATACCACTGGGGCATACTTCATGC

PLRV-sgRNA : CTTGAGCCTCGTCCTCGGGGAACTCCAG

PVY-sgRNA : TTGGTTTTGCATTCTCAACGATTGGTTT

Supplementary Figure 1. The architecture of *PTG* gene for producing four sgRNAs. sgRNAs are 100% complementarity to the *CP* genes of PVS, PVX, PLRV and PVY. Stem-loop structure: the scaffold region of the sgRNA for interaction with Cas13d. Poly(A): a signal for sgRNA exporting out of the nucleus.

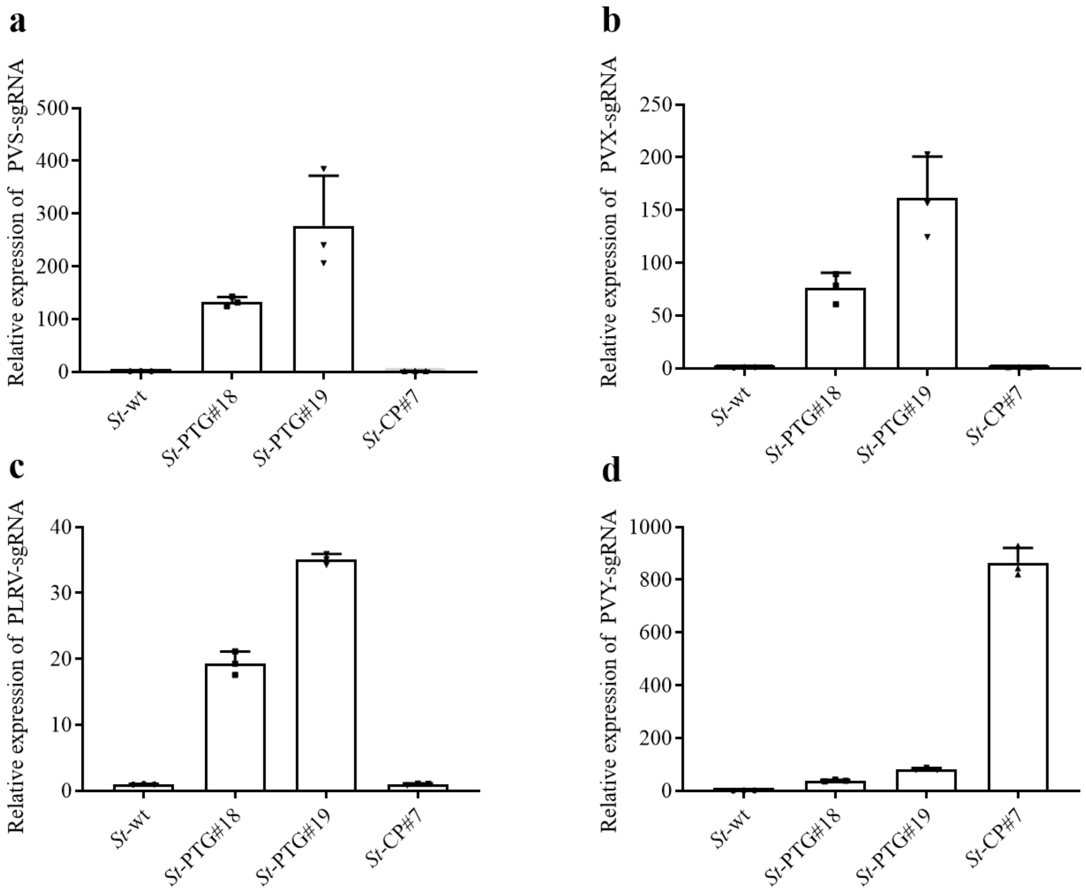

Supplementary Figure 2. Analysis of the relative expression of four gRNAs in the transgenic lines by qRT-PCR. The *TUBULIN2* gene was used as internal standard. Note that the expression of four gRNAs were all detected in the transgenic lines *St*-PTG#18 and *St*-PTG#19, while only the presence of PVY-sgRNA was detected in the transgenic line *St*-CP#7 plant. Data are showed as means ± SD (n = 3).

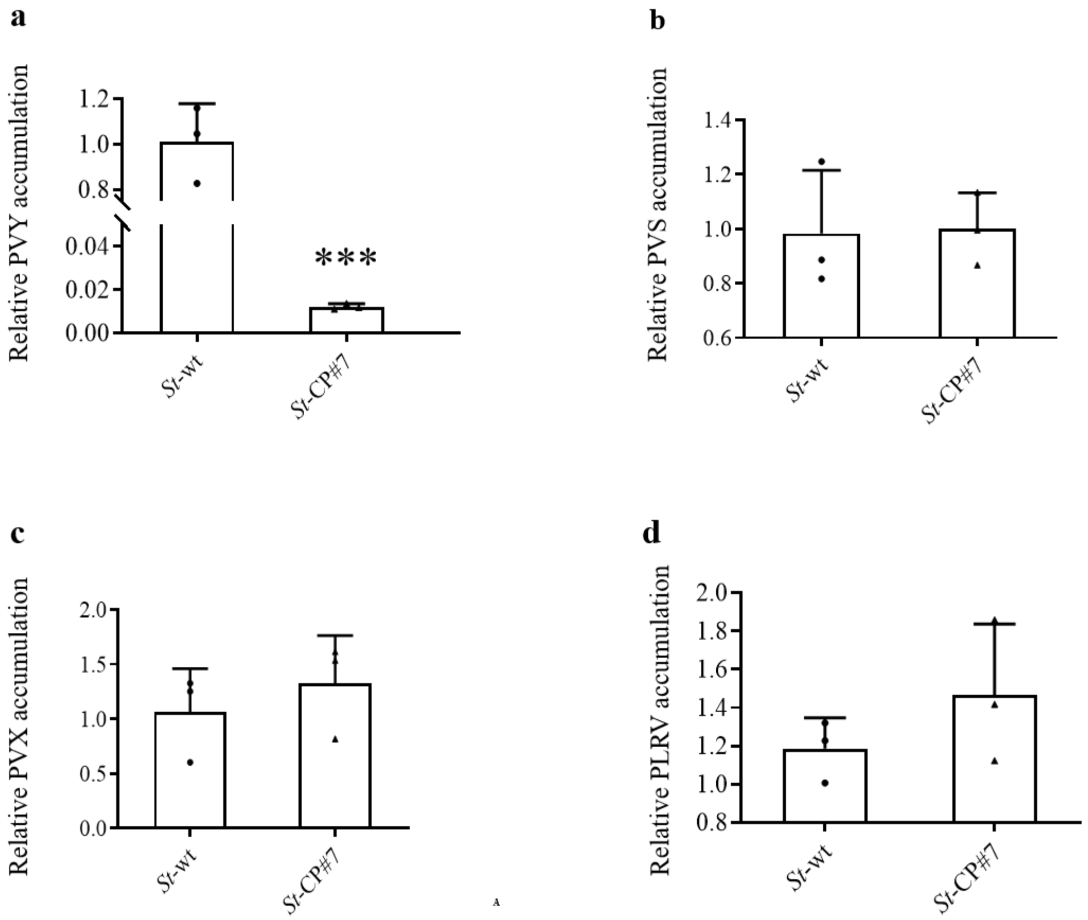

Supplementary Figure 3. Transgenic potato plants (*St*-CP#7) targeting the *CP* gene of PVY (*St*-CP#7) displays specific resistance to PVY (**a**), while not to PVS (**b**), PVX (**c**) or PLRV (**d**). Virus accumulation were assayed by qRT-PCR. The data represent as means ± SD (n = 3). Asterisks indicate statistically significant differences (***P < 0.001, independent-samples t-test).

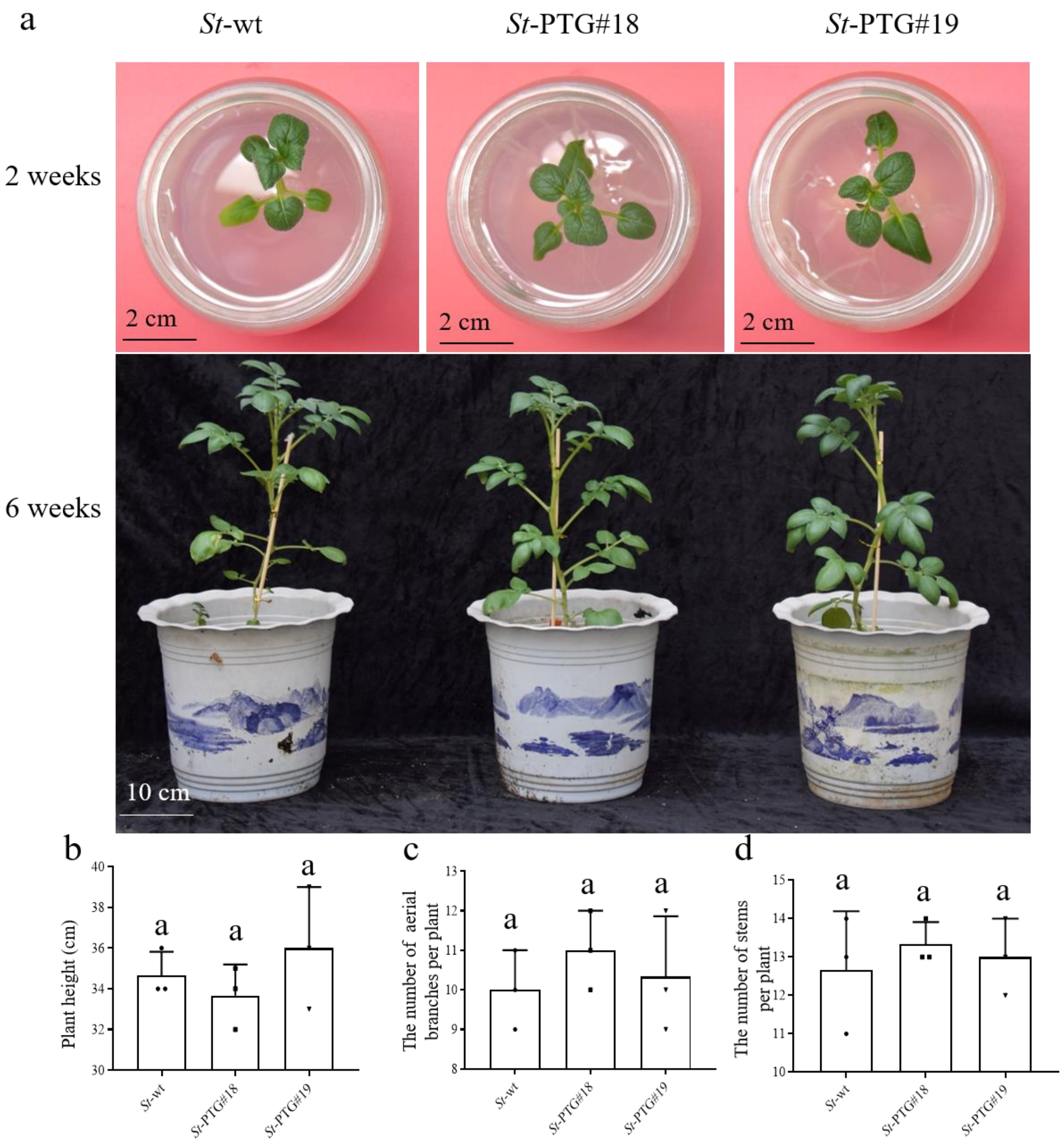

Supplementary Figure 4. Phenotypic characterization of the transgenic plants. (**a**) The growth and phenotypes of transgenic and wild type plants under mixotrophic condition (grown in MS medium) for 2 weeks (upper panel) and autotrophic condition (grown in soil) for 6 weeks (lower panel). Plant height (**b**), the number of aerial branches per plant (**c**) and the number of stems per plant (**d**) of 6-week old plants grown in soil. The data represent as means ± SD (n = 3). No significant differences among the transgenic and wild-type plants were determined by one-way ANOVA (P > 0.05).

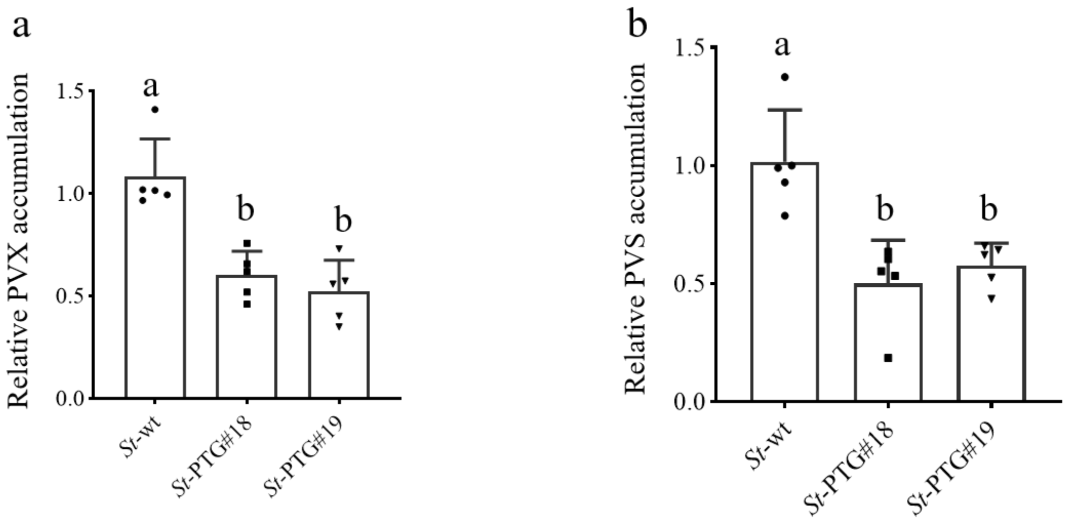

Supplementary Figure 5. The resistance of the transgenic plants (*St*-PTG#18 and *St*-PTG#19) to PVX (**a**) or PVS (**b**) infection at 15 dpi. Virus accumulation of PVX and PVS was assayed by qRT-PCR. Data are means ± SD, and represent five biological replicates (n = 5). The letters above the bar indicate the significant difference of multiple comparison with one-way ANOVA ($P < 0.05$).

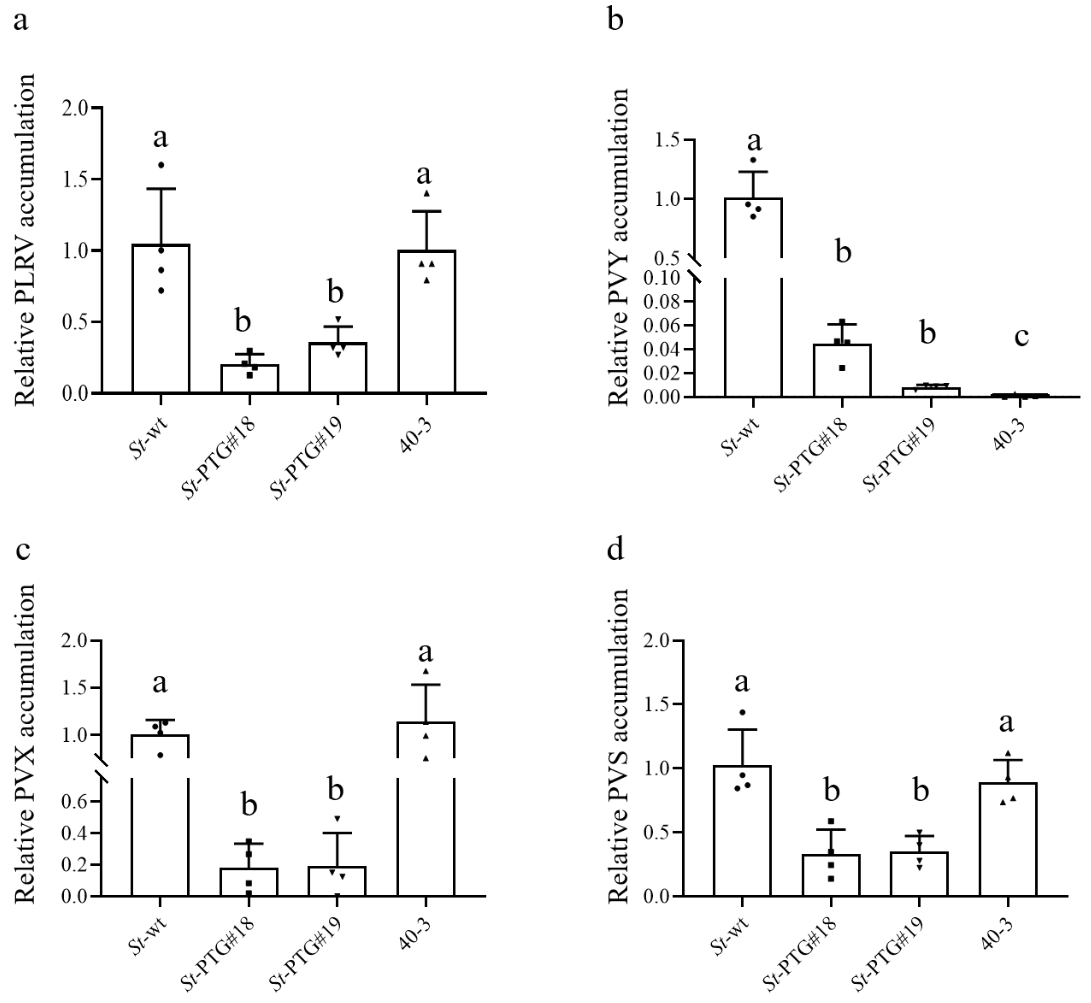

Supplementary Figure 6. The resistance level of the transgenic plants (*St*-PTG#18 and *St*-PTG#19) to PLRV (**a**), PVY (**b**), PVX (**c**) or PVS (**d**) infection when compared to the PVY-resistant *S. chacoense* accession 40-3. Virus accumulation of PLRV (**a**), PVY (**b**), PVX (**c**) or PVS (**d**) was assayed by qRT-PCR. Data are means ± SD, and represent four biological replicates (n = 4). The letters above the bar indicate the significant difference of multiple comparison with one-way ANOVA ($P < 0.05$).

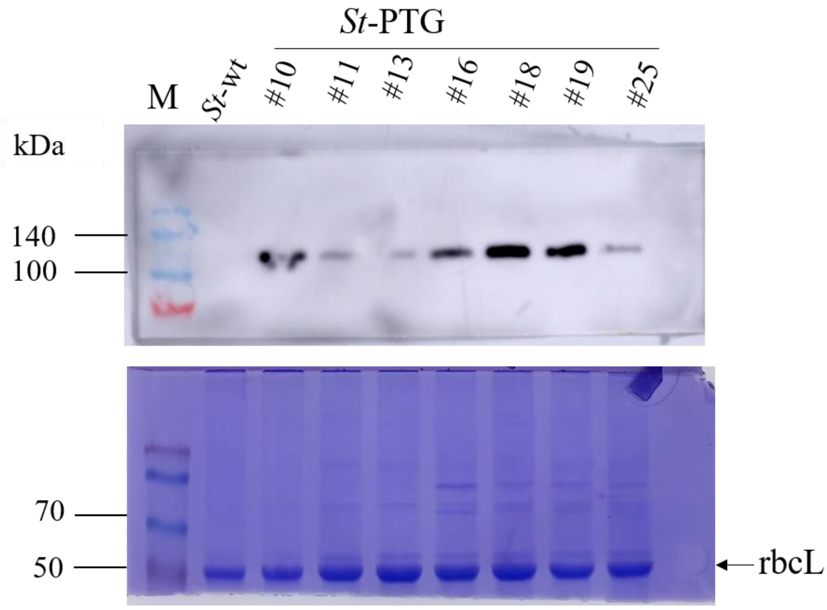

Supplementary Figure 7. Uncropped and unedited blot/gel for western blot analysis of the Cas13d expression in the transgenic potato lines. Coomassie staining of another identical gel is shown below the protein blot as a control for equal loading (10 μg of total soluble protein). The large subunit of Rubisco (rbcL), the most abundant protein in leaves, in indicated.

Supplementary Note 1: Codon-optimized *Cas13d* sequence.

ATGATCGAGAAGAAGAAGTCCTTCGCCAAAGGCATGGGAGTGAAGTCTACTCTCGTGAG
CGGAAGCAAGGTGTACATGACTACTTTCGCTGAGGGATCTGATGCCAGGCTCGAAAAGAT
CGTTGAGGGCGATTCTATCCGTAGCGTGAACGAAGGTGAAGCTTTCTCTGCTGAGATGG
CTGACAAGAACGCTGGATACAAGATCGGGAACGCCAAGTTCTCTCACCCTAAGGATATG
CTGTGGTGGCTAACAACCCTCTCTACACTGGACCTGTTCAGCAGGATATGCTCGGGCTCA
AAGAGACTCTCGAGAAGAGATACTTCGGGGAGTCTGCTGATGGGAACGACAACATCTGT
ATCCAGGTGATCCACAACATCCTCGACATTGAGAAGATCCTCGCCGAGTACATCACCAAC
GCTGCTTACGCTGTGAACAACATCTCTGGACTCGACAAGGACATCATCGGCTTCGGAAA
GTTCTCTACCGTGTACACCTACGACGAGTTCAAGGATCCTGAGCATCACAGGGCTGCCTT
CAACAACAACGACAAGCTCATCAACGCTATCAAGGCCCAGTACGATGAGTTCGATAACTT
CCTCGATAACCCGAGGCTCGGTTATTTCGGACAGGCCTTCTTTAGCAAAGAGGGCCGTAA
CTACATCATCAACTACGGGAACGAGTGCTACGACATCCTCGCTTTGCTTTCTGGACTCAG
GCATTGGGTCGTGCATAACAACGAGGAAGAGAGCAGAATCTCTAGGACCTGGCTCTACA
ACCTCGACAAGAACCTTGACAACGAGTACATTAGCACCCTCAACTACCTCTACGACAGGA
TCACTAACGAGCTGACCAACAGCTTCAGCAAGAACAGCGCTGCTAACGTGAACTACATTG
CTGAGACACTCGGAATCAACCCGGCTGAATTTGCTGAGCAGTACTTCAGGTTCAGCATCA
TGAAGGAACAGAAGAACCTCGGGTTCAACATCACGAAGCTCCGTGAGGTTATGCTCGAC
CGTAAGGACATGTCTGAGATCCGTAAGAACCACAAGGTGTTCGACAGCATCAGGACCAA
GGTCTACACCATGATGGACTTCGTGATCTACAGGTACTACATCGAAGAGGACGCTAAGGT
GGCAGCCGCTAACAAGTCTCTTCCAGACAACGAGAAGTCGCTCAGCGAGAAGGACATCT
TCGTTATCAACCTCCGTGGCAGCTTCAACGATGACCAAAAGGATGCTCTCTACTACGATG
AGGCCAACAGGATCTGGCGTAAGCTCGAGAACATCATGCACAACATCAAAGAGTTCCGA
GGGAACAAGACCCGTGAGTACAAGAAGAAGGACGCTCCTAGACTCCCTAGGATTCTTCC
TGCTGGTAGAGATGTGAGCGCCTTCTCTAAGCTTATGTACGCCCTCACCATGTTCCTCGA
CGGGAAAGAGATCAACGACCTCCTCACTACCCTCATCAACAAGTTCGACAACATTCAGTC
GTTCCTCAAGGTGATGCCTCTCATCGGAGTGAACGCTAAGTTCGTTGAAGAGTACGCCTT
CTTCAAGGACAGCGCTAAGATCGCTGATGAGCTGAGGCTCATCAAGAGCTTCGCTAGAAT
GGGAGAGCCTATCGCAGATGCTAGAAGGGCTATGTACATCGACGCTATCCGTATCCTCGG
GACCAACCTTTCTTACGACGAGCTTAAGGCTCTCGCCGATACTTTCAGCCTTGACGAGAA
CGGAAACAAGCTCAAGAAAGGGAAGCACGGCATGAGGAACTTCATTATCAACAACGTGAT
CAGCAACAAGAGGTTCCACTACCTCATCCGTTACGGTGATCCTGCTCATCTCCATGAGAT
CGCTAAGAACGAGGCTGTGGTTAAGTTCGTGCTCGGACGTATCGCTGACATCCAGAAAAA
GCAAGGACAGAACGGGAAGAACCAGATCGACCGTTACTACGAGACTTGCATCGGAAAGG
ACAAGGGCAAGTCCGTGTCTGAGAAAGTTGATGCGCTCACCAAGATCATCACCGGGATG
AACTACGACCAGTTTGACAAGAAGCGTAGCGTCATCGAGGATACCGGAAGAGAAAATGCT
GAGCGTGAGAAGTTCAAGAAGATCATCTCCCTCTACCTCACGGTGATCTACCATATCCTCA
AGAACATCGTGAACATCAACGCCCGTTACGTCATCGGATTCCACTGCGTTGAAAGAGATG
CCCAGCTCTACAAAGAGAAGGGGTACGACATCAACCTCAAGAAGCTCGAGGAAAAGGGG
TTCAGCTCTGTGACTAAGCTTTGCGCTGGAATCGATGAGACTGCTCCTGATAAGAGGAAG
GACGTCGAAAAAGAGATGGCCGAGAGGGCCAAAGAGTCTATCGACTCTCTTGAGAGCGC
TAACCCTAAGCTCTACGCCAACTACATTAAGTACTCGGATGAGAAGAAGGCCGAAGAGTT
CACCAGGCAGATCAACAGAGAGAAGGCTAAGACTGCTCTCAACGCCTACCTCAGAAACA
CCAAGTGGAACGTGATAATCCGTGAGGACCTCCTCCGTATCGATAACAAGACTTGCACCC

TGTTCCGTAACAAGGCTGTGCATCTCGAGGTGGCAAGATATGTGCACGCTTACATCAACG
ATATCGCCGAGGTCAACAGCTACTTCCAGCTGTACCACTACATTATGCAGAGGATCATCAT
GAACGAGAGGTACGAGAAGAGTAGCGGCAAGGTGTCAGAGTACTTCGATGCTGTGAACG
ACGAAAAGAAGTACAACGACCGACTCCTCAAGCTCCTCTGTGTTCCTTTCGGATACTGCA
TCCCGCGTTTCAAGAACCTCTCTATCGAGGCTCTCTTCGATAGAAACGAGGCGGCCAAAT
TCGACAAAGAAAAGAAGAAAGTCAGCGGGAACTCTTACCCATACGATGTTCCAGATTACG
CTTATCCTTATGACGTACCTGATTATGCATACCCGTACGATGTGCCTGACTACGCGTGA

Supplementary Note 2：Sequence of *PTG* gene.

AACAAAGCACCAGTGGTCTAGTGGTAGAATAGTACCCTGCCACGGTACAGACCCGGGTTCG
ATTCCCGGCTGGTGCACCACCCCAATATCGAAGGGGACTAAAACCTCTTGTGAGCGTTATG
AGCTATCGTCTAAAAAAAAACAAAGCACCAGTGGTCTAGTGGTAGAATAGTACCCTGCCAC
GGTACAGACCCGGGTTCGATTCCCGGCTGGTGCACCACCCCAATATCGAAGGGGACTAAAA
CTTCCATACCACTGGGGCATACTTCATGCAAAAAAAAACAAAGCACCAGTGGTCTAGTGGT
AGAATAGTACCCTGCCACGGTACAGACCCGGGTTCGATTCCCGGCTGGTGCACCACCCCAA
TATCGAAGGGGACTAAAACCTTGAGCCTCGTCCTCGGGGAACTCCAGAAAAAAAAACAAA
GCACCAGTGGTCTAGTGGTAGAATAGTACCCTGCCACGGTACAGACCCGGGTTCGATTCCC
GGCTGGTGCACCACCCCAATATCGAAGGGGACTAAAACTTGGTTTTGCATTCTCAACGATT
GGTTTAAAAAAA

The sequences are annotated as follows:
Grey letters present tRNA sequence; yellow letters present stem-loop sequence; red letters present PVS-sgRNA spacer; green letters present PVX-sgRNA spacer; blue letters present PLPV-sgRNA spacer; purple letters present PVY-sgRNA spacer.

Supplementary Table 1. List of oligonucleotides used in this study.

Oligonucleotide	Sequence 5'-3'	Description and Use
Tubulin2-F	GATGTTGTGCCAAAGGATGT	forward primer for qRT-PCR analysis of *TUBULIN* expression
Tubulin2-R	AACTTGTGGTCAATGCGAGA	reverse primer for qRT-PCR analysis of *TUBULIN* expression
Cas13d-qPCR-F	GACTTGCACCCTGTTCCGTA	forward primer for qRT-PCR analysis of Cas13d expression
Cas13d-qPCR-R	GCCGCTACTCTTCTCGTACC	reverse primer for qRT-PCR analysis of Cas13d expression
PVY-3S	ACGTCCAAAATGAGAATGCC	forward primer for qRT-PCR analysis of PVY^{O}
PVY-R1	GCGAGGTTCCATTTTCAATGCA	reverse primer for qRT-PCR analysis of PVY^{O}
PLRV-qPCR-F	TTGCCTCCTGGAACTCCGTGAA	forward primer for qRT-PCR analysis of PLRV
PLRV-qPCR-R	AGCGTCCAATAAAGACCGAACCAAA	reverse primer for qRT-PCR analysis of PLRV
PVX-qPCR-F	AAGCCTGAGCACAAATTCGC	forward primer for qRT-PCR analysis of PVX
PVX-qPCR-R	GCTTCAGACGGTGGCCG	reverse primer for qRT-PCR analysis of PVX
PVS-qPCR-F	TGTCGAGGGGCTAATCCGTA	forward primer for qRT-PCR analysis of PVS
PVS-qPCR-R	GCATTCCGATGGTTACGCAC	reverse primer for qRT-PCR analysis of PVS
sgRNA-F	ACCACCCCAATATCGAAGGGGACTA	forward primer for qRT-PCR analysis of sgRNA expression
PVS-CP-sgRNA-R	AGACGATAGCTCATAACGCTCACA	reverse primer for qRT-PCR analysis of PVS-CP expression
PVX-CP-sgRNA-R	GCATGAAGTATGCCCCAGTGG	reverse primer for qRT-PCR analysis of PVX-CP expression
PLRV-CP-sgRNA-R	CTGGAGTTCCCCGAGGACGAG	reverse primer for qRT-PCR analysis of PLRV-CP expression
PVY-CP-sgRNA-R	CCAATCGTTGAGAATGCAAAAC	reverse primer for qRT-PCR analysis of PVY-CP expression
