# Supplementary material for: Cas13d-mediated multiplex RNA targeting confers a broad-spectrum resistance against RNA viruses in potato
Source: Commun Biol. 2023 Aug 17;6:855. doi: 10.1038/s42003-023-05205-2 (PMC10435558; doi:10.1038/s42003-023-05205-2)
Supplement: Supplementary file 4 — Reporting Summary [file 42003_2023_5205_MOESM4_ESM.pdf]

nature portfolio

Corresponding author(s): Jiang Zhang and Fengjuan Zhang

Last updated by author(s): Jul 20, 2023

Reporting Summary

Nature Portfolio wishes to improve the reproducibility of the work that we publish. This form provides structure for consistency and transparency in reporting. For further information on Nature Portfolio policies, see our Editorial Policies and the Editorial Policy Checklist.

Statistics

For all statistical analyses, confirm that the following items are present in the figure legend, table legend, main text, or Methods section.

n/a	Confirmed	
☐	☒	The exact sample size (*n*) for each experimental group/condition, given as a discrete number and unit of measurement
☐	☒	A statement on whether measurements were taken from distinct samples or whether the same sample was measured repeatedly
☐	☒	The statistical test(s) used AND whether they are one- or two-sided<br>*Only common tests should be described solely by name; describe more complex techniques in the Methods section.*
☒	☐	A description of all covariates tested
☒	☐	A description of any assumptions or corrections, such as tests of normality and adjustment for multiple comparisons
☐	☒	A full description of the statistical parameters including central tendency (e.g. means) or other basic estimates (e.g. regression coefficient) AND variation (e.g. standard deviation) or associated estimates of uncertainty (e.g. confidence intervals)
☒	☐	For null hypothesis testing, the test statistic (e.g. *F*, *t*, *r*) with confidence intervals, effect sizes, degrees of freedom and *P* value noted<br>*Give P values as exact values whenever suitable.*
☒	☐	For Bayesian analysis, information on the choice of priors and Markov chain Monte Carlo settings
☒	☐	For hierarchical and complex designs, identification of the appropriate level for tests and full reporting of outcomes
☒	☐	Estimates of effect sizes (e.g. Cohen's *d*, Pearson's *r*), indicating how they were calculated

Our web collection on statistics for biologists contains articles on many of the points above.

Software and code

Policy information about availability of computer code

Data collection	GraphPad 8.0 was used to prepare the data, IBM SPASS Statistics 19 was used to analysis all data
Data analysis	Statistical analyses of the data were performed by t tests for comparison of two groups and one-way analysis of variance (ANOVA) coupled with Tukey and Dunnett's test for multiple comparisons.

For manuscripts utilizing custom algorithms or software that are central to the research but not yet described in published literature, software must be made available to editors and reviewers. We strongly encourage code deposition in a community repository (e.g. GitHub). See the Nature Portfolio guidelines for submitting code & software for further information.

Data

Policy information about availability of data

All manuscripts must include a data availability statement. This statement should provide the following information, where applicable:

- Accession codes, unique identifiers, or web links for publicly available datasets
- A description of any restrictions on data availability
- For clinical datasets or third party data, please ensure that the statement adheres to our policy

Cas13d (Ruminococcus flavefaciens XPD3002, CasRx), PVYO-FL (HM367075), PLRV (MT264739.1), PVX (AB056718.1) and PVS (KU896946)

Research involving human participants, their data, or biological material

Policy information about studies with human participants or human data. See also policy information about sex, gender (identity/presentation), and sexual orientation and race, ethnicity and racism.

Reporting on sex and gender	not include in our study
Reporting on race, ethnicity, or other socially relevant groupings	not include in our study
Population characteristics	not include in our study
Recruitment	not include in our study
Ethics oversight	not include in our study

Note that full information on the approval of the study protocol must also be provided in the manuscript.

Field-specific reporting

Please select the one below that is the best fit for your research. If you are not sure, read the appropriate sections before making your selection.

☒ Life sciences ☐ Behavioural & social sciences ☐ Ecological, evolutionary & environmental sciences

For a reference copy of the document with all sections, see nature.com/documents/nr-reporting-summary-flat.pdf

Life sciences study design

All studies must disclose on these points even when the disclosure is negative.

Sample size	No sample size calculation was performed, which were based on our experiences in performing similar experiments and published studies. And sample sizes are available for each figure.
Data exclusions	no data were excluded from the ananlysis
Replication	to verify the reproducibility of the experimental findings , every data was represent three technical repeats, and every experiments were showed five biological replicates ,and repeat at least three times
Randomization	the samples were allocated randomly
Blinding	No blinding used because the disease symptoms were clearly distinguishable at the first sight

Reporting for specific materials, systems and methods

We require information from authors about some types of materials, experimental systems and methods used in many studies. Here, indicate whether each material, system or method listed is relevant to your study. If you are not sure if a list item applies to your research, read the appropriate section before selecting a response.

Materials & experimental systems

n/a	Involved in the study	
☐	☒	Antibodies
☒	☐	Eukaryotic cell lines
☒	☐	Palaeontology and archaeology
☒	☐	Animals and other organisms
☒	☐	Clinical data
☒	☐	Dual use research of concern
☐	☒	Plants

Methods

n/a	Involved in the study	
☒	☐	ChIP-seq
☒	☐	Flow cytometry
☒	☐	MRI-based neuroimaging

Antibodies

Antibodies used	anti-HA antibody (ABclonal, AE008) , HRP Goat Anti-Mouse IgG (ABclonal, AS003)

Validation

anti-HA antibody (ABclonal) https://abclonal.com.cn/catalog/AE008
HRP Goat Anti-Mouse IgG (H+L) (ABclonal) https://abclonal.com.cn/catalog/AS003
